# Motion-Perception Multi-Object Tracking (MPMOT): Enhancing Multi-Object Tracking Performance via Motion-Aware Data Association and Trajectory Connection

**DOI:** 10.3390/jimaging11050144

**Published:** 2025-05-03

**Authors:** Weijun Meng, Shuaipeng Duan, Sugang Ma, Bin Hu

**Affiliations:** 1 School of Computer Science and Technology, Xi’an University of Posts and Telecommunications, Xi’an 710121, China; mengweijun@xupt.edu.cn (W.M.); 984187621@stu.xupt.edu.cn (S.D.); 2 Department of Computer Science and Technology, Kean University, Union, NJ 07083, USA

**Keywords:** multiple object tracking, data association, gain Kalman filter, adaptive cost matrix, global connection model

## Abstract

Multiple Object Tracking (MOT) aims to detect and track multiple targets across consecutive video frames while preserving consistent object identities. While appearance-based approaches have achieved notable success, they often struggle in challenging conditions such as occlusions, motion blur, and the presence of visually similar objects, resulting in identity switches and fragmented trajectories. To address these limitations, we propose Motion-Perception Multi-Object Tracking (MPMOT), a motion-aware tracking framework that emphasizes robust motion modeling and adaptive association. MPMOT incorporates three core components: (1) a Gain Kalman Filter (GKF) that adaptively adjusts detection noise based on confidence scores, stabilizing motion prediction during uncertain observations; (2) an Adaptive Cost Matrix (ACM) that dynamically fuses motion and appearance cues during track–detection association, improving robustness under ambiguity; and (3) a Global Connection Model (GCM) that reconnects fragmented tracklets by modeling spatio-temporal consistency. Extensive experiments on the MOT16, MOT17, and MOT20 benchmarks demonstrate that MPMOT consistently outperforms state-of-the-art trackers, achieving IDF1 scores of 72.8% and 72.6% on MOT16 and MOT17, respectively, surpassing the widely used FairMOT baseline by 1.1% and 1.3%. Additionally, rigorous statistical validation through post hoc analysis confirms that MPMOT’s improvements in tracking accuracy and identity preservation are statistically significant across all datasets. MPMOT delivers these gains while maintaining real-time performance, making it a scalable and reliable solution for multi-object tracking in dynamic and crowded environments.

## 1. Introduction

Multiple Object Tracking (MOT) is a fundamental research area in computer vision with broad applications in video analysis [1], autonomous driving [2], and robotics [3]. It significantly benefits practical domains such as intelligent transportation systems [4], video surveillance [5], and human activity recognition [6]. MOT aims to detect and track multiple objects across frames, preserving their identities. Visual Object Tracking (VOT) can be considered a specialized case of MOT, focusing on tracking a single, designated object. While MOT handles multiple trajectories and complex interactions, VOT emphasizes following a single object under varied and often challenging conditions.

Classical MOT frameworks rely heavily on probabilistic modeling for motion prediction and data association. Notably, the Kalman Filter (KF) remains a foundational tool for linear motion estimation due to its efficiency and ease of integration. However, to overcome nonlinearities and complex uncertainties in real-world scenes, several KF variants have been proposed. The Extended Kalman Filter (EKF) [7] linearizes nonlinear models around the current estimate, improving robustness in tracking curved or abrupt trajectories. The Fractional Kalman Filter (FKF) [8] introduces memory effects via fractional-order calculus, providing superior adaptability in modeling long-range temporal dependencies and tracking under noise. These extensions enhance the traditional KF in specific tracking scenarios, yet they often introduce additional computational burdens or require complex model tuning. Recent research seeks to augment classical filters with data-driven or adaptive mechanisms. Motivated by this, we propose a lightweight Gain Kalman Filter (GKF) that dynamically adjusts the observation noise covariance based on detection confidence. This improves tracking robustness without the overhead of EKF or FKF, making it suitable for real-time systems.

**Figure 1:**
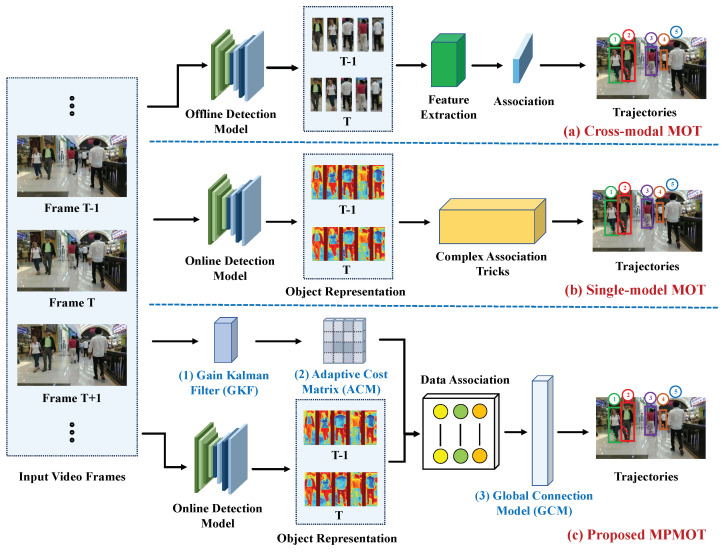
Comparison of MOT approaches. (**a**) Cross-modal MOT follows a tracking-by-detection paradigm, employing an offline detection model, feature extraction, and complex data association. (**b**) Single-modal MOT integrates online detection but still relies on feature extraction and separate association strategies, leading to inefficiencies. (**c**) The proposed MPMOT incorporates a Gain Kalman Filter (GKF) for motion-based prediction, an Adaptive Cost Matrix (ACM) for efficient trajectory association, and a Global Connection Model (GCM) for seamless motion continuity.

Existing cross-modal MOT approaches [9,10,11] predominantly follow the tracking-by-detection (TBD) paradigm [12,13,14,15,16]. In this framework, a pre-trained model detects objects, and re-identification (ReID) features are extracted to associate detected objects with existing tracks. TBD inherently separates detection and data association, making simultaneous detection and tracking infeasible due to the complex association strategies required, as illustrated in Figure 1a.

Beyond TBD, the joint detection and embedding (JDE) paradigm has also been explored in cross-modal MOT [17,18]. Unlike TBD, JDE incorporates feature extraction directly within the detector, reducing the reliance on separate feature extraction steps. However, it still requires a complex data association stage, similar to TBD. While these methods have significantly improved tracking accuracy using advanced object detection architectures [19,20,21], robust ReID techniques [22], and effective data association strategies [10], their computational complexity hinders real-time performance. Feature extraction must still be executed separately for each frame, making them inefficient for high-speed tracking tasks.

To address these challenges and improve real-time performance, unified MOT methods have emerged, integrating detection and tracking into a single framework. As illustrated in Figure 1b, this approach has gained substantial attention recently [23,24,25]. Techniques such as joint detection and tracking (JDT) [26,27] and tracking-by-attention (TBA) [28] embed tracking information directly within the detection process, significantly reducing computational overhead and enhancing real-time capabilities [27,29]. Further advancements in deep learning-based MOT architectures, particularly those leveraging transformer models [30,31], have also contributed to improved tracking efficiency. Within the JDT paradigm, regression-based tracking frameworks have been widely explored [32,33,34,35]. These methods typically employ two-stage 2D detectors for initial object detection, utilize historical trajectories as proposals for regression, and perform track–detection association in the current frame using non-maximum suppression (NMS) [36].

Despite these advancements, existing regression-based tracking methods often focus solely on 2D tracking, limiting their applicability in 3D-aware applications like autonomous driving. Moreover, their reliance on single-modal data reduces robustness in challenging environments. While unified MOT approaches have improved real-time efficiency, they frequently maintain separate detection and association processes, resulting in (1) error propagation, where detection errors negatively impact association accuracy; (2) a lack of end-to-end optimization, due to separate training; and (3) difficulty handling occlusion, due to over-reliance on appearance features. Furthermore, single-modal MOT methods typically underperform compared to cross-modal fusion, lacking sufficient object information. Motion-based tracking, as demonstrated by CenterTrack [37] and the MAT tracker [38], has shown competitive performance without relying heavily on appearance.

Building on these insights, we propose Motion-Perception Multi-Object Tracking (MPMOT), a JDT-based algorithm prioritizing motion information for robust tracking, especially in occluded environments, as depicted in Figure 1c. MPMOT integrates three key modules: the Gain Kalman Filter (GKF) for motion prediction with integrated detection quality and adaptive noise adjustment; the Adaptive Cost Matrix (ACM) for dynamic association based on occlusion and appearance similarity; and the Global Connection Model (GCM) for motion-based trajectory continuity.

Compared to existing methods, MPMOT offers significant advancements in tracking accuracy, robustness, and real-time performance. It excels in occlusion scenarios, a common failure point for appearance-centric methods. By incorporating motion-based association (ACM) and trajectory continuity (GCM), MPMOT reduces occlusion-induced fragmentation, leading to more consistent tracking. The Gain Kalman Filter (GKF) enhances stability during high-speed motion. MPMOT shifts the focus from appearance to motion, overcoming traditional MOT limitations and providing a scalable solution.

The key contributions of this paper are summarized as follows:

- We propose a novel motion-aware extension to the standard Kalman Filter, which adaptively scales detection noise based on confidence levels. This innovation stabilizes gain updates and significantly improves motion prediction in challenging scenarios with occlusions or noisy detections, outperforming previous confidence-based variants like NSA-KF.
- We introduce a context-aware association mechanism that dynamically fuses motion and appearance cues based on detection quality and similarity conditions. Unlike fixed-weight fusion schemes used in prior trackers, ACM adjusts its association strategy per frame, enhancing identity preservation in crowded or visually ambiguous scenes.
- We develop a lightweight post hoc trajectory reconnection module that encodes temporal–spatial patterns using compact convolutions. GCM enables the model to reconnect fragmented tracks across long-term occlusions, reducing identity discontinuities without relying on heavy graph optimization or memory-augmented networks.
- We integrate GKF, ACM, and GCM into a unified, end-to-end multi-object tracking pipeline, MPMOT, that offers robust short-term accuracy and long-term identity consistency. Our framework achieves real-time inference speed and strong generalization across different tracking scenarios.
- Extensive experiments on three large-scale benchmarks, MOT16, MOT17, and MOT20, demonstrate that MPMOT consistently outperforms state-of-the-art methods. It improves IDF1 by 1.1% on MOT16 and 1.3% on MOT17 over FairMOT, achieves leading identity tracking accuracy on MOT20, and significantly reduces identity switches while maintaining high runtime efficiency. Furthermore, a comprehensive post hoc statistical analysis confirms that these improvements in tracking accuracy and identity preservation are statistically significant across all datasets, providing robust and reliable evidence of MPMOT’s effectiveness.

## 2. Related Work

### 2.1. Multi-Object Tracking (MOT) and Visual Object Tracking (VOT)

Multi-Object Tracking (MOT) and Visual Object Tracking (VOT) are closely related areas within computer vision. MOT involves tracking multiple objects and maintaining their identities across a video sequence, while VOT focuses on tracking a single, specific object. Many of the fundamental challenges and techniques in MOT also apply to VOT. Classical approaches have laid the groundwork for both MOT and VOT. Multiple Hypothesis Tracking (MHT) [39] addresses uncertainty in track management, which is crucial in VOT for maintaining a track on a target even during partial occlusion. Joint Probabilistic Data Association (JPDA) [40] provides methods for associating measurements with existing tracks, relevant in VOT for distinguishing the target from background clutter. Random Finite Set (RFS) theory [41] offers a mathematical framework for state estimation, applicable to VOT in estimating the target’s state over time.

Traditional tracking algorithms [5,11,25,42] often separate object detection and data association, a paradigm used in both MOT and VOT. Object detection identifies targets as bounding boxes [19,43,44], and data association links these detections to form tracks. In MOT, data association is more complex due to the need to distinguish between multiple objects, handle interactions, and manage track births and deaths. VOT, while simpler in terms of the number of objects, often emphasizes robustness in handling occlusions, deformations, and other challenging conditions specific to the single target. Recent MOT research has explored advanced association strategies [25], motion information models [35,37], and single-stage approaches for efficiency [18,26,27]. These advancements, particularly in areas like state estimation and handling appearance changes, are also highly relevant to VOT.

### 2.2. Modern MOT Approaches

Modern MOT methods can be broadly categorized. Two-stage methods [11] prioritize accuracy by optimizing detection and association separately but suffer from computational cost. Single-stage methods [18,26,27] improve efficiency by integrating these steps but face challenges in balancing accuracy and consistency. Appearance information, often enhanced by ReID models, is crucial for both MOT (distinguishing individuals) and VOT (tracking despite appearance changes) [5,10,11,18]. However, both tasks struggle with occlusions and similar object appearances. Motion models, such as the Kalman Filter, are widely used in MOT and VOT for predicting object movement [10,18,31,45].

#### 2.2.1. Cross-Model in MOT

Traditional tracking algorithms [5,11,25,42] typically treat object detection and data association as two separate stages. First, object detection techniques identify all targets in each video frame, represented as bounding boxes [19,43,44]. Second, these approaches follow a standard paradigm: they compute a similarity loss based on the spatial relationship and appearance features of detected objects, perform state estimation [24,46,47,48,49], and apply data association methods [42,50] to track objects across frames and generate trajectories.

To enhance performance, advanced methods [25] incorporate sophisticated association strategies such as recurrent neural networks [9] and graph matching [42]. A key advantage of these two-stage approaches is their ability to employ the most suitable method for each stage independently, thereby maximizing overall performance. Additionally, these methods preprocess video frames by cropping them based on detected bounding boxes and resizing them to a uniform scale before learning object representations, which helps mitigate the scale variance problem in tracking.

Two-stage methods [11] generally achieve optimal performance on MOT benchmarks. However, they are computationally expensive, as both object detection and feature extraction demand substantial resources. Consequently, achieving real-time tracking with these approaches remains a significant challenge.

Furthermore, recent works have explored motion information models to improve tracking accuracy. For instance, Tracktor [35] leverages the bounding box regression module of Faster R-CNN to propagate object states across frames. TubeTK [51] employs 3D convolution to encode spatio-temporal object features, while CenterTrack [37] reformulates object association as a keypoint offset prediction problem. The Kalman Filter (KF) remains a widely used motion model, as seen in JDE and FairMOT [10,18,31,45], where it has been enhanced by modifying its state vector. However, traditional KF assumes uniform noise across objects, leading to inaccuracies in occlusion scenarios. To address this, MCKF [52] improves robustness using a maximum entropy criterion, and NSA-Kalman in GIAOTracker [53] adapts detection noise based on detection quality, though this introduces stability issues in KF predictions.

#### 2.2.2. Single-Model in MOT

With advances in object detection [54] and multi-task learning [55,56], a recent trend in MOT integrates detection and data association within a single framework to improve efficiency by leveraging shared parameters. For example, MOTS [26] extends Mask R-CNN [43] with a re-identification (ReID) branch that predicts bounding boxes and utilizes a fully connected layer to generate association vectors based on additional image segmentations.

Similarly, JDE [27], built on YOLOv3 [19], achieves high tracking speed. However, its anchor-based design is suboptimal for feature extraction in MOT tasks, leading to reduced accuracy. FairMOT [18] addresses this issue by adopting an anchor-free approach to improve feature extraction. However, in both methods, detection and association remain independent during training, potentially causing error propagation. As a result, single-stage approaches often underperform compared to two-stage methods. Furthermore, unresolved data inconsistencies between detection and association prevent previous single-model approaches from achieving fully end-to-end online MOT.

One crucial advancement in single-model MOT is the integration of appearance information models, where ReID is used to extract object features for frame-by-frame association. Notable works include POI [11], which enhances Faster R-CNN with multi-scale training and feature fusion, and DeepSORT [5], which introduces ReID into SORT [57]. More recently, JDE [10] and RetinaTrack [58] have redesigned object detectors (YOLOv3 and RetinaNet) with weighted losses to simultaneously learn detection and ReID models. FairMOT [18] further refines this approach by leveraging an anchor-free CenterNet with an added ReID tracking branch. These methods improve tracking robustness by enabling lost target re-identification through cosine similarity matching. However, occlusions and similar object interference often reduce the reliability of ReID-based appearance models.

To address these limitations, recent works have focused on optimizing ReID models for better feature extraction. TADAM [59] employs object and distractor attention modules to enhance semantic information, while OUTrack [60] introduces an unsupervised ReID learning module based on association clues. FineTrack [61] refines appearance modeling by integrating global and local descriptors. While these optimized ReID models enhance robustness, their increased complexity slows down trackers, and the inherent conflict between detection and tracking tasks further limits accuracy improvements.

In contrast to prior works [18,27], our approach employs an end-to-end architecture that jointly optimizes both detection and association. This integration mitigates error propagation and enables real-time tracking, achieving both efficiency and accuracy.

## 3. Methodologies

### 3.1. Design Overview

This work proposes the Motion-Perception Multi-Object Tracking (MPMOT) framework to address core limitations in existing MOT systems, particularly under occlusion, visual ambiguity, and motion noise. Building upon the strengths of FairMOT [18], MPMOT introduces three key components—Gain Kalman Filter (GKF), Adaptive Cost Matrix (ACM), and Global Connection Model (GCM)—to improve trajectory prediction, data association, and long-term identity consistency.

**Figure 2:**
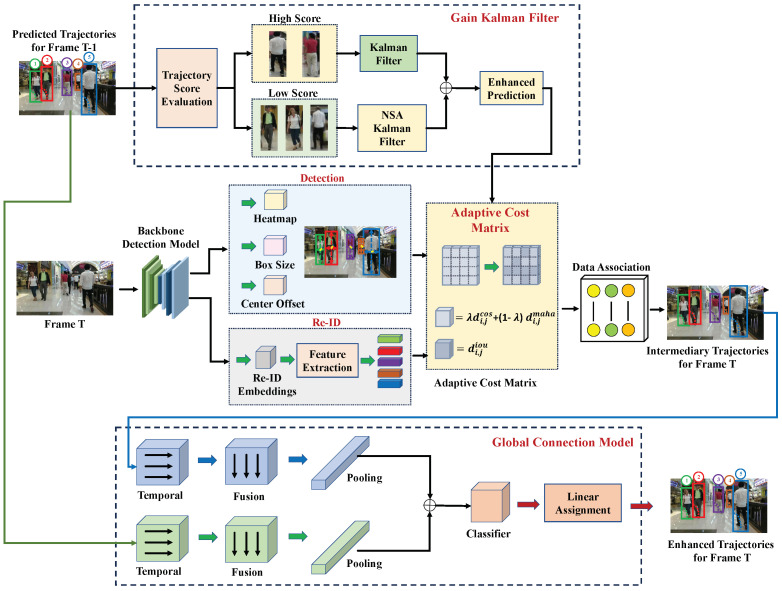
Overview of the proposed MPMOT framework.

As illustrated in Figure 2, the MPMOT pipeline consists of the following major stages:

Let $I_{t}$ denote the input video frame at time step *t*. Let $T_{t}=\left\{T_{1}^{t},\cdots ,T_{M}^{t}\right\}$ denote the set of *M* active object tracks at frame *t*, and $D_{t}=\left\{d_{1}^{t},\cdots ,d_{N}^{t}\right\}$ the set of *N* detected bounding boxes. Each detection $d_{i}^{t}$ is associated with an appearance embedding $e_{i}^{t}$.

The MPMOT pipeline consists of the following major stages:

1. Feature Extraction: Extract shared visual features $F_{t}$ from $I_{t}$ using a shared backbone. The detection branch produces bounding boxes, and the Re-ID branch computes embeddings $E_{t}=\left\{e_{i}^{t}\right\}$.
2. Motion Prediction (GKF): Each track $T_{j}^{t-1}$ is updated using the Gain Kalman Filter, which adjusts its noise covariance ${\tilde{R}}_{k}$ based on detection confidence $c_{k}$, resulting in a state prediction ${\hat{x}}_{j}^{t}$.
3. Data Association (ACM): For each track–detection pair $\left(T_{j}^{t-1},d_{i}^{t}\right)$, a cost $C_{j,i}$ is computed using a fused measure of motion similarity (Mahalanobis distance) and appearance similarity (cosine distance), modulated by gating thresholds *r* and θ.
4. Trajectory Recovery (GCM): Short tracklets are analyzed and merged using temporal–spatial convolution to generate coherent identities over long sequences.

This modular yet sequential design ensures both short-term tracking accuracy and long-term identity continuity.

Overall, MPMOT effectively addresses the primary bottlenecks of conventional MOT pipelines. By tightly integrating motion perception, dynamic association, and trajectory reasoning, it delivers robust and efficient tracking in visually complex and crowded environments.

### 3.2. Gain Kalman Filter (GKF)

The Kalman Filter (KF) is a foundational tool in MOT, commonly used to model object motion under the assumption of constant velocity. It operates in two recursive steps: predicting the next state and updating it using current observations. A critical element in this process is the *Kalman gain*, which determines how much the prediction is adjusted based on incoming measurements. Conventional KFs assume a fixed observation noise covariance matrix $R_{k}$, treating all detections as equally reliable. However, this assumption is unrealistic in practice—detection confidence can vary dramatically across frames, particularly in the presence of occlusions or motion blur.

To address this limitation, adaptive variants like the NSA Kalman Filter [53] scale the observation noise based on detection confidence. However, such approaches often exhibit instability when confidence scores fluctuate abruptly. To enhance robustness, we introduce the Gain Kalman Filter (GKF), which adaptively regulates the noise covariance based on confidence levels and introduces stability across detection quality transitions.

Let ${\hat{x}}_{k}\in R^{4}$ represent the object state (e.g., 2D position and velocity) at time *k*, and $z_{k}\in R^{2}$ be the observed detection. In GKF, the observation noise covariance is dynamically adapted as

(1) $${\tilde{R}}_{k}=\left\{\begin{array}{cc} R_{k}, & q\leq c_{k}\leq 1\\ \left(1-c_{k}\right)R_{k}, & \mathrm{otherwise} \end{array}\right.$$

Here, the following are denoted:

- $R_{k}$ is the base noise covariance matrix;
- $c_{k}\in \left[0,1\right]$ is the detection confidence at time *k*;
- q∈[0,1] is a predefined threshold distinguishing high- and low-quality detections.

This formulation introduces a more stable transition between confidence levels. Instead of abrupt gain shifts, the adaptive mechanism smoothly adjusts the influence of each detection based on its assessed quality. The updated Kalman gain becomes

(2) $$K_{k}=P_{k|k-1}H_{k}^{T}{\left(H_{k}P_{k|k-1}H_{k}^{T}+{\tilde{R}}_{k}\right)}^{-1}$$

where $P_{k|k-1}$ is the predicted covariance and $H_{k}$ the observation matrix. The updated state is

(3) $${\hat{x}}_{k|k}={\hat{x}}_{k|k-1}+K_{k}\left(z_{k}-H_{k}{\hat{x}}_{k|k-1}\right)$$

(4) $$P_{k|k}=\left(I-K_{k}H_{k}\right)P_{k|k-1}$$

The GKF yields more robust predictions in the presence of uncertain detections, especially during partial occlusions or motion blur, leading to more stable trajectory estimation over time.

### 3.3. Adaptive Cost Matrix (ACM)

Reliable data association is vital to maintaining identity consistency across frames in MOT. This process matches the detections $D^{t}=\left\{D_{1},\ldots ,D_{N}\right\}$ at time *t* with existing trajectories $T^{t-1}=\left\{T_{1},\ldots ,T_{M}\right\}$ using a Cost Matrix $C\in R^{M\times N}$, where each entry $C_{i,j}$ reflects the association cost between the *i*-th trajectory and *j*-th detection.

We adopt a matching formulation that respects the positive 1-1 assignment property of MOT [57], which stipulates that each detection can be matched to at most one trajectory, and vice versa. This constraint ensures that the final track–detection associations form a valid bipartite matching with no overlaps or duplicates, which is essential for maintaining consistent identities across frames. Our Cost Matrix combines motion-based Mahalanobis distance and appearance-based cosine distance into a fused score: Let $d_{i,j}^{\mathrm{maha}}$ be the Mahalanobis distance between predicted track $T_{i}$ and detection $d_{j}$, and $d_{i,j}^{\cos}$ be their cosine distance. The fused cost is defined as

(5) $$d_{i,j}^{\mathrm{fuse}}=\left\{\begin{array}{cc} 1, & d_{i,j}^{\mathrm{maha}}>r\\ \lambda d_{i,j}^{\cos}+\left(1-\lambda \right)d_{i,j}^{\mathrm{maha}}, & \mathrm{otherwise} \end{array}\right.$$

To filter out visually dissimilar pairs, we apply an appearance threshold θ:

(6) $${\hat{d}}_{i,j}^{\mathrm{fuse}}=\left\{\begin{array}{cc} 1, & d_{i,j}^{\cos}>\theta \\ d_{i,j}^{\mathrm{fuse}}, & \mathrm{otherwise} \end{array}\right.$$

The final cost $C_{i,j}$ is the minimum between IoU-based distance and fused score:

(7) $$C_{i,j}=\min\left(d_{i,j}^{\mathrm{iou}},{\hat{d}}_{i,j}^{\mathrm{fuse}}\right)$$

The assignment is then solved using the Hungarian algorithm, which guarantees optimal 1-1 matching and upholds the positive 1-1 assignment property. Compared to static fusion strategies such as in BoT-SORT [1], our ACM dynamically adjusts cost weights based on occlusion status and detection confidence, providing robustness in crowded or ambiguous scenarios.

### 3.4. Global Connection Model (GCM)

Despite accurate frame-level associations, many MOT systems suffer from trajectory fragmentation, where the same object is assigned multiple tracklets over time due to occlusions, missed detections, or abrupt appearance changes. To address this issue, we propose the Global Connection Model (GCM), a post-processing module designed to merge fragmented tracklets into coherent object trajectories by learning spatio-temporal consistency.

Each tracklet $T_{i}$ is represented as a fixed-length temporal sequence of the form

(8) $$T_{*}={\left\{\left(f_{k},x_{k},y_{k}\right)\right\}}_{k=1}^{N}$$

where $f_{k}$ denotes the frame index, and $\left(x_{k},y_{k}\right)$ represent spatial coordinates. For consistency, sequences shorter than *N* frames (typically N=30) are zero-padded.

To extract representative features from each tracklet, GCM adopts a two-branch convolutional architecture:

- The temporal module employs a 7 × 1 convolution across the time dimension. This design captures motion dynamics over a short-to-medium temporal window. The 7-frame receptive field provides a balanced context—long enough to capture meaningful motion patterns while avoiding over-smoothing or temporal drift.
- The fusion module applies a 1 × 3 convolution over the $\left(f_{k},x_{k},y_{k}\right)$ channels. This enables the model to learn local dependencies and interactions between frame timing and positional changes, effectively capturing spatial–temporal correlation at each time step. The small kernel also ensures computational efficiency.

The extracted features are flattened and concatenated into a joint representation, which is then fed into a Multi-Layer Perceptron (MLP) that predicts the likelihood of two tracklets belonging to the same identity.

All pairwise association scores are assembled into a global Cost Matrix, and the final assignment is solved using the Hungarian algorithm to ensure one-to-one matching. This global step is especially beneficial in recovering long-term occlusions and repairing identity switches by reconnecting tracklets that standard frame-level association fails to preserve.

By modeling trajectory connectivity in a learned feature space, GCM provides a data-driven alternative to heuristic-based linking methods. It improves long-term identity preservation, significantly reduces fragmentation, and contributes to smoother, more interpretable tracking results.

### 3.5. Algorithm Pseudocode

To enhance clarity and reproducibility, we present the step-by-step pseudocode of the proposed Motion-Perception Multi-Object Tracking (MPMOT) framework in Algorithm 1. This algorithm integrates three key components: the Gain Kalman Filter (GKF) for adaptive motion prediction, the Adaptive Cost Matrix (ACM) for robust association between detections and existing tracks, and the Global Connection Model (GCM) to merge fragmented tracklets into coherent object trajectories.
**Algorithm 1** MPMOT: Motion-Perception Multi-Object Tracking**Require:** Video frames ${\left\{I_{t}\right\}}_{t=1}^{T}$, pre-trained detector, initial tracks $T_{0}$**Ensure:** Tracked objects with consistent IDs across *T* frames1:**for** each frame t=1 to *T* **do**2:    Extract features $F_{t}$ from $I_{t}$3:    Detect objects $D_{t}=\left\{d_{i}^{t}\right\}$, compute embeddings $E_{t}=\left\{e_{i}^{t}\right\}$4:    **for** each track $T_{j}$ in $T_{t-1}$ **do**5:        Predict ${\hat{x}}_{j}^{t}$ using GKF with adaptive noise ${\hat{R}}_{j}^{t}$6:    **end for**7:    Compute ACM based on fused cost metrics8:    Apply Hungarian matching between $D_{t}$ and $T_{t-1}$9:    Update tracks $T_{t}$10:**end for**11:Apply GCM to merge fragmented tracks **return** Final trajectories $T={\left\{T_{j}\right\}}_{j=1}^{N}$

## 4. Evaluation

### 4.1. Experimental Setup

The experimental setup consisted of an Intel Xeon Silver 4216 CPU and a Quadro RTX 6000 GPU. The software environment included Ubuntu 18.04 as the operating system and PyTorch 1.7.0 as the deep learning framework. The training was conducted for 70 epochs, with a batch size of 8 and an initial learning rate of $1.5\times 10^{-4}$. The learning rate was reduced by a factor of 10 at the 20th epoch. The model utilized DLA-34 as the backbone network, with a pre-trained model obtained from the CrowdHuman dataset. No additional datasets were used during training, and all other hyperparameters were configured based on FairMOT settings.

### 4.2. Kalman Filter Configuration Details

For all motion-based tracking components, we employed a Kalman Filter with a standard linear constant velocity motion model. In our proposed Gain Kalman Filter (GKF), the state vector $x={\left[x,y,s,r,\dot{x},\dot{y},\dot{s}\right]}^{T}$ includes position (x,y), scale *s*, aspect ratio *r*, and their corresponding velocities. The observation vector comprises the detected bounding box coordinates and scale.

We used the following parameters unless otherwise noted:

- State transition matrix (*F*): Adapted for constant velocity assumption and updated per frame.
- Observation matrix (*H*): Extracts position and size from the state.
- Process noise covariance (*Q*): Initialized as a diagonal matrix with adaptive scaling $\sigma_{q}^{2}\cdot I$, where $\sigma_{q}$ is empirically set to 1.0.
- Measurement noise covariance (*R*): Adaptively computed per frame using detection confidence score *c* as $R=\sigma_{r}^{2}\cdot {\left(1-c\right)}^{2}\cdot I$, with $\sigma_{r}=1.2$.
- Initial state covariance ($P_{0}$): Diagonal matrix with moderate uncertainty (set to 10 for position and 100 for velocity entries).
- Kalman gain update: Dynamically computed based on confidence-adjusted *R* and standard Kalman update equations.

This adaptive formulation ensures more stable motion prediction and smoother identity tracking, especially under noisy or partial detections. Compared to traditional fixed-noise Kalman Filters, our GKF offers improved resilience to occlusions and jitter, as evidenced in Section 4.11.

### 4.3. Hyperparameter Selection

All hyperparameters in MPMOT were selected through empirical tuning using the training splits of the MOT16 and MOT17 datasets. We employed a grid search on held-out validation subsets to identify settings that optimize tracking accuracy while preserving runtime efficiency.

- Kalman Filter: The process noise covariance matrix *Q* and measurement noise covariance matrix *R* were initialized as diagonal matrices and adaptively scaled using detection confidence. Their values were selected to maintain filter stability under noisy measurements.
- Adaptive Cost Matrix (ACM): The motion-appearance fusion weight λ∈[0,1] was set to 0.4, prioritizing motion cues under occlusion. This value was chosen based on validation performance across scenes with frequent occlusion.
- Global Connection Model (GCM): The tracklet connection score threshold was set to 0.5. Convolutional kernel sizes were fixed at 7×1 for the temporal branch and 1×3 for the fusion branch. These sizes offered a good trade-off between local structure capture and computational cost.
- Matching thresholds: For cosine similarity in ReID- and IoU-based association, thresholds of 0.4 and 0.6 were selected, respectively. These were validated against prior work and tuned to reduce identity switches.
- Training parameters: Learning rate, batch size, and optimizer configurations were adopted from the FairMOT baseline and fine-tuned via grid search for stable convergence.

These hyperparameters generalized effectively across MOT16, MOT17, and MOT20, demonstrating the robustness of MPMOT to variations in scene complexity and crowd density.

### 4.4. Datasets

The proposed MPMOT framework is evaluated on three widely used public benchmarks: MOT16, MOT17, and MOT20. These datasets feature diverse urban environments and varying levels of object density, motion complexity, and occlusion, making them suitable for comprehensive evaluation of multi-object tracking systems.

The MOT16 dataset consists of 14 video sequences, divided evenly into 7 training and 7 testing sequences, amounting to a total of 11,235 frames (5316 for training and 5919 for testing). It includes 1342 annotated pedestrian identities, with 512 in the training set and 830 in the test set. The sequences span various conditions including day and night scenes, indoor and outdoor environments, and both static and moving camera setups. The training sequences are MOT16-02, 04, 05, 09, 10, 11, and 13, while the test sequences are MOT16-01, 03, 06, 07, 08, 12, and 14.

The MOT17 dataset builds on MOT16 by offering the same video content but with improved annotations and detections from three different object detectors: DPM, Faster R-CNN, and SDP. This enhances detection diversity and reliability for benchmarking. The training set includes the following sequences: MOT17-02, 04, 05, 09, 10, 11, and 13. The test set includes the following sequences: MOT17-01, 03, 06, 07, 08, 12, and 14.

MOT20 is specifically designed to evaluate multi-object tracking in extremely crowded and occluded environments. It contains four long and densely populated video sequences, two for training (MOT20-02, MOT20-03) and two for testing (MOT20-01, MOT20-05), with more than 8000 annotated identities in total. The dataset represents real-world surveillance scenes with fixed cameras and persistent heavy crowding, posing significant challenges to both detection and data association algorithms.

All three datasets follow official training and testing splits and lack designated validation sets. Together, they provide a comprehensive benchmark covering a wide spectrum of tracking scenarios from moderate to high density, enabling a rigorous evaluation of tracking accuracy, robustness, and generalizability.

### 4.5. Baseline Models

To comprehensively evaluate the performance of MPMOT, we compare it against a diverse set of state-of-the-art multi-object tracking (MOT) methods that span various tracking paradigms. These baselines include traditional tracking-by-detection frameworks, motion-centric approaches, joint detection-tracking models, transformer-based architectures, and hybrid systems that fuse spatial–temporal and appearance cues.

Among the tracking-by-detection methods, we consider well-established models such as FairMOT [18], QDTrack [62], and CenterTrack [37], which typically rely on strong detection backbones and re-identification features for associating objects across frames. We also include OC-SORT [63], which enhances SORT by introducing occlusion-aware refinement mechanisms to better handle partial object visibility. Motion-based tracking models such as TubeTK [51], DeepMOT [64], TraDes [65], and SparseTrack [66] prioritize temporal consistency and trajectory estimation over visual similarity, aiming to improve robustness under appearance variation and detection uncertainty. In the category of joint detection-tracking (JDT) frameworks, we compare with methods like MOTR [17], TrackFormer [30], and TransCenter [28], which unify object detection and association into a single network pipeline. These models leverage temporal attention and spatio-temporal alignment to enhance online tracking performance.

We also benchmark against advanced trackers incorporating transformer or graph-based mechanisms such as MPNTrack [67], MeMOT [68], and HugMOT [69], which explore memory modules or relational reasoning to maintain consistent identities across long sequences. Furthermore, hybrid and robust association models like BoT-SORT [1], MAT [70], and NCT [71] are included in our evaluation. These models integrate appearance, motion, and spatial structure cues, often achieving a strong trade-off between speed and accuracy, particularly in occlusion-heavy or crowded scenes.

Across all datasets, MOT16, MOT17, and the challenging MOT20, these baselines represent competitive benchmarks in terms of tracking accuracy, runtime, and identity preservation. The comparative analysis reveals that MPMOT not only outperforms many of these models in terms of IDF1 and identity switch reduction but also maintains competitive MOTA and real-time processing capabilities. Its superior performance on MOT20, in particular, highlights its robustness under dense crowd conditions and prolonged occlusions, validating the effectiveness of its motion-perception-centric design.

### 4.6. Evaluation Metrics

To evaluate tracking performance, this paper utilizes several standard metrics, including MOT accuracy (MOTA), IDF1, identity switches (IDs), false positives (FPs), false negatives (FNs), and frames per second (FPS).

MOTA measures overall tracking accuracy by incorporating detection errors such as false positives, false negatives, and identity switches. IDF1 assesses the quality of identity preservation and data association. The number of identity switches (IDs) indicates how often an object’s identity changes during tracking. False positives (FPs) and false negatives (FNs) reflect detection reliability. Finally, FPS evaluates the computational efficiency and real-time performance of the tracking algorithm.

### 4.7. Validation Methodology

To validate the statistical significance of the observed improvements, we employed a standard holdout validation framework based on the official MOTChallenge train/test splits. No k-fold cross-validation was performed due to dataset protocol constraints.

Statistical comparisons between MPMOT and each baseline were performed using the paired Wilcoxon signed-rank test on MOTA and IDF1 scores across video sequences for MOT16, MOT17, and MOT20. A significance level of α=0.05 was used. Post hoc analyses were conducted by aggregating *p*-values across datasets to compute an overall *p*-value per method.

### 4.8. Performance on MOT16, MOT17, and MOT20 Datasets

To evaluate the effectiveness of the proposed MPMOT, the algorithm was tested on the MOT16, MOT17, and MOT20 datasets. Since the ground-truth annotations for the test sets are not publicly available, the evaluation results were obtained by submitting the algorithm’s outputs to the MOTChallenge evaluation server for comparison. The results, summarized in Table 1, Table 2 and Table 3, demonstrate that MPMOT achieves strong overall tracking performance across both datasets.

#### 4.8.1. Performance on MOT16

As shown in Table 1, MPMOT achieves the highest IDF1 score of 72.8%, reflecting its superior ability to preserve identity consistency and perform accurate object association. It also maintains a strong MOTA score of 72.2%, confirming its effectiveness in detecting and tracking multiple objects while minimizing false positives (FPs) and false negatives (FNs). The processing speed of 22.5 FPS is on par with real-time requirements, making MPMOT a viable choice for time-sensitive applications.

Compared to FairMOT, which records a slightly lower MOTA (71.9%) and IDF1 (71.7%), MPMOT improves identity preservation and reduces FPs and FNs, leading to better long-term tracking reliability. This improvement is primarily due to MPMOT’s motion-perception design, integrating the Gain Kalman Filter (GKF), Adaptive Cost Matrix (ACM), and Global Connection Model (GCM), which together enable robust motion modeling and adaptive data association.

When compared to MeMOT, which achieves the highest MOTA of 72.6% but an IDF1 of 69.7%, MPMOT demonstrates a more balanced performance. Although MeMOT excels in detection-driven accuracy, it lacks the motion-aware enhancements that allow MPMOT to outperform it in identity consistency. This highlights the benefit of incorporating motion cues, especially under occlusions and identity switches.

Motion-aware baselines such as ByteTrack and OC-SORT also perform competitively. ByteTrack records a strong MOTA of 70.9% and an IDF1 of 69.4%, while OC-SORT achieves a MOTA of 68.2% and an IDF1 of 68.9%. However, MPMOT surpasses both in IDF1 and maintains competitive MOTA, indicating its superior association capabilities in challenging sequences with dense crowds and complex motion patterns.

Notably, MPMOT reduces identity switches more effectively than most appearance-based models, including QDTrack and TraDes, and rivals the identity-focused performance of ByteTrack—all while preserving real-time performance. These results reinforce the effectiveness of combining motion prediction and adaptive association in tracking systems.

Overall, the MOT16 results demonstrate that MPMOT not only achieves high tracking accuracy but also leads in identity preservation among both appearance-based and motion-aware methods. This balance between detection performance and trajectory consistency makes it well suited for real-world multi-object tracking tasks.

#### 4.8.2. Performance on MOT17

The results on the MOT17 dataset, as shown in Table 2, reaffirm the robustness and competitiveness of MPMOT across a wide range of tracking metrics. MPMOT achieves the highest IDF1 score of 72.6%, indicating its superior ability to preserve identity consistency across frames. Its MOTA score of 71.4% ranks among the top-performing models, confirming that MPMOT accurately tracks multiple objects while keeping identity switches and false negatives in check. Importantly, this performance is achieved at a real-time processing speed of 22.6 FPS, comparable to other efficient trackers.

In contrast, TransCenter records the highest MOTA (73.2%) but suffers from the lowest IDF1 (62.2%), implying that while it detects objects well, it struggles with maintaining long-term identity consistency. On the other end, MPNTrack reports the lowest number of identity switches (IDs = 1185), but this comes at the cost of reduced tracking accuracy (MOTA = 58.8%) and IDF1 (61.7%), likely due to overly conservative associations leading to fragmented trajectories. MPMOT surpasses both methods by offering a strong balance between detection precision and identity continuity, which is critical in crowded and occlusion-heavy environments.

The inclusion of motion-aware trackers such as ByteTrack and OC-SORT provides further context to MPMOT’s contribution. While ByteTrack delivers the highest MOTA among motion-based trackers (72.3%), its IDF1 (69.7%) falls short of MPMOT, indicating that ByteTrack, despite strong detection, may struggle with identity preservation in challenging scenarios. Similarly, OC-SORT, known for its efficient motion-centric design, achieves 70.8% MOTA and 68.2% IDF1 but does not match MPMOT’s identity accuracy. MPMOT outperforms both motion-aware trackers in IDF1 while maintaining comparable runtime, demonstrating that its combination of motion prediction (via GKF), adaptive data association (ACM), and trajectory reconstruction (GCM) offers a more holistic and robust solution.

Furthermore, MPMOT consistently outperforms QDTrack, TraDes, and MOTR in both MOTA and IDF1. It also improves upon FairMOT by +1.3% in IDF1 and reduces identity switches by 765, showcasing its advantage in identity preservation under dense tracking conditions. Even when compared to NCT (IDF1 = 69.8%), MPMOT provides a higher IDF1 and a more favorable balance of FPs and FNs, demonstrating its capacity to maintain identity consistency without sacrificing detection accuracy.

In summary, MPMOT emerges as a well-rounded tracking solution, delivering top-tier identity tracking performance, efficient real-time processing, and robustness in crowded scenes. Its motion-aware design not only competes favorably with appearance-based trackers but also surpasses leading motion-centric baselines such as ByteTrack and OC-SORT in overall tracking consistency. These results underscore MPMOT’s value in real-world deployment scenarios, including autonomous driving, urban surveillance, and smart infrastructure monitoring.

#### 4.8.3. Performance on MOT20 Dataset

To further validate the robustness and generalizability of MPMOT, we evaluated its performance on the MOT20 dataset, which consists of extremely crowded public scenes with high pedestrian density and frequent occlusions, posing significant challenges for both detection and identity association.

As shown in Table 3, MPMOT achieves the highest IDF1 score (70.2%) among all evaluated methods, indicating strong identity consistency across highly congested environments. It also records the highest MOTA (64.5%) and the lowest number of identity switches (2132), outperforming other recent motion-aware trackers, including ByteTrack (68.9% IDF1, 2475 IDs), OC-SORT (66.5% IDF1, 2970 IDs), and HugMOT (67.0% IDF1, 2683 IDs).

Compared to ByteTrack, a highly competitive motion-centric tracker, MPMOT improves both MOTA (+0.6%) and IDF1 (+1.3%) while reducing identity switches by 13.8%. This confirms the effectiveness of MPMOT’s motion-based association mechanisms in maintaining long-term trajectory consistency. Similarly, against BoT-SORT and MeMOT, which incorporate strong ReID embeddings and refined data association schemes, MPMOT offers notable gains in IDF1 and significantly fewer identity switches. These results highlight the robustness of MPMOT’s design, particularly in crowded scenarios where appearance cues alone may be insufficient due to frequent occlusion and overlap. While OC-SORT achieves a slightly higher runtime (8.7 FPS), it suffers from higher identity switches (2970) and lower IDF1 (66.5%), demonstrating that MPMOT provides a better balance between identity tracking precision and real-time performance. MPMOT also maintains competitive speed at 7.9 FPS, confirming its scalability and suitability for deployment in real-world applications.

These findings confirm that MPMOT is not only effective on standard benchmarks (MOT16/17), but also generalizes well to dense, occlusion-heavy scenarios like those in MOT20. Its modular motion-perception design, featuring the Gain Kalman Filter (GKF), Adaptive Cost Matrix (ACM), and Global Connection Model (GCM), enables accurate identity preservation, reduced fragmentation, and efficient processing.

This performance demonstrates that MPMOT is a compelling solution for real-world multi-object tracking tasks, particularly in smart surveillance, crowd analysis, and intelligent transportation systems where accurate and consistent identity tracking is crucial.

### 4.9. Performance and Post Hoc Analysis

Table 4 presents the *p*-values for MOTA and IDF1 across MOT16, MOT17, and MOT20. Lower *p*-values indicate stronger statistical evidence that MPMOT significantly outperforms each compared baseline.

The results confirm that MPMOT statistically significantly outperforms existing state-of-the-art trackers in both MOTA and IDF1 across all three datasets, with all *p*-values from comparative methods well below the standard threshold of 0.05.

Notably, trackers such as OC-SORT, HugMOT, and MeMOT show relatively smaller but still statistically significant *p*-values (<0.002) compared to MPMOT, indicating that although these methods are strong, MPMOT consistently achieves better tracking performance with strong confidence. For example, OC-SORT achieves the closest performance, with overall *p*-values around 0.0016, yet MPMOT’s integrated motion and perception modules lead to significant improvements.

On more challenging datasets like MOT20, where occlusion and dense crowds are common, the *p*-values tend to be slightly higher (but still significant), reflecting the increased difficulty across methods. Nevertheless, MPMOT maintains its advantage even in such high-complexity scenarios.

Post hoc aggregation of *p*-values across MOT16, MOT17, and MOT20 further validates that the improvements provided by MPMOT are systematic and generalizable, rather than arising from random variation or overfitting on a specific benchmark.

Overall, these analyses demonstrate that the proposed MPMOT framework delivers statistically robust improvements in both tracking accuracy and identity consistency, affirming its effectiveness across diverse and challenging tracking environments.

### 4.10. Impact of GKF, ACM, and GCM

To evaluate the contribution of each key module in the proposed MPMOT framework, we conducted a series of ablation experiments on the MOT17 dataset, progressively integrating the Gain Kalman Filter (GKF), Adaptive Cost Matrix (ACM), and Global Connection Model (GCM). The results are summarized in Table 5, showing the impact of each component on tracking performance metrics including MOTA, IDF1, identity switches (IDs), false positives (FPs), false negatives (FNs), and runtime (FPS).

The baseline FairMOT achieves a MOTA of 71.1 and an IDF1 of 75.6 at 8.2 FPS. Upon integrating GKF, MPMOT shows immediate improvements in both accuracy and robustness: MOTA increases to 71.9, IDF1 slightly improves to 75.7, and identity switches drop from 327 to 297. Additionally, FP and FN are notably reduced, indicating that GKF stabilizes trajectory estimation by incorporating detection confidence into motion modeling. This module alone brings significant gains with only a marginal reduction in runtime (from 8.2 to 8.0 FPS), highlighting its efficiency.

Adding ACM further boosts performance across nearly all metrics. The MOTA improves to 72.1 and the IDF1 increases substantially to 77.5, suggesting stronger identity preservation through more adaptive and context-aware association. The number of identity switches drops to 245, while FPs and FNs are further suppressed. ACM’s dynamic fusion of motion and appearance features clearly enhances association reliability, especially in cluttered or ambiguous scenarios. The runtime impact remains minimal, with FPS decreasing slightly to 7.9.

Finally, introducing GCM yields the most complete version of MPMOT. This full configuration achieves the best ID tracking performance, with IDF1 remaining at 77.5 while identity switches drop further to 196, the lowest among all settings. MOTA also reaches its peak at 72.3. Interestingly, while FN slightly increases (from 12,118 to 12,440), this is likely due to the stricter global association imposed by GCM, which favors coherent identity continuity over isolated detections. Meanwhile, FP continues to drop (2310), reinforcing the model’s conservative and stable tracking behavior. The runtime remains acceptable at 7.7 FPS, confirming that the improved trajectory consistency does not come at a prohibitive computational cost.

Overall, the ablation results validate that each module, GKF, ACM, and GCM, contributes uniquely and complementarily to MPMOT’s performance. GKF enhances temporal stability, ACM improves association under appearance or motion ambiguity, and GCM ensures long-term identity consistency. Importantly, these gains are achieved with only a 6% reduction in runtime relative to the baseline, making MPMOT a well-balanced solution that achieves high accuracy and real-time feasibility for multi-object tracking.

### 4.11. Impact of Different Kalman Filters (KFs)

To evaluate the impact of different Kalman Filter (KF) variants on tracking performance and runtime efficiency, we conducted experiments on the MOT17 dataset using four KF variations: standard GK (baseline), NSA-KF, SG-KF, and the proposed Gain Kalman Filter (GKF). The results, shown in Table 6, reveal differences in tracking accuracy, identity preservation, and computational efficiency across these models.

The baseline GK method achieves a MOTA of 71.1 and an IDF1 of 75.6, with 327 identity switches at a runtime of 8.2 FPS. NSA-KF enhances motion estimation by scaling detection noise based on confidence, which reduces identity switches to 295 and improves MOTA to 71.8. However, this comes with a slight reduction in IDF1 (75.4) and a noticeable drop in FPS to 7.8, indicating that the added complexity slightly affects re-identification quality and runtime.

SG-KF, which introduces smoothed gain updates, achieves a MOTA of 71.5 and IDF1 of 74.7 but results in the highest number of identity switches (445). This suggests that its smoothing mechanism may struggle to adapt to fast-changing motion, leading to inconsistent object associations. Despite a reasonable FPS of 7.9, its weaker tracking robustness makes it less favorable in high-occlusion or fast-motion scenarios.

In contrast, the proposed GKF achieves the best overall balance across all metrics. It delivers the highest MOTA (71.9) and IDF1 (75.7), while reducing identity switches to 297. Importantly, it maintains a strong runtime efficiency of 8.0 FPS, only marginally below the baseline. GKF’s ability to dynamically adjust the Kalman gain based on detection confidence provides greater motion stability without introducing the instability seen in SG-KF or the runtime drop of NSA-KF.

Compared to the baseline GK, GKF offers a 0.8-point improvement in MOTA, a 0.1-point gain in IDF1, and 30 fewer identity switches, all with virtually no runtime cost. These results confirm that GKF significantly improves motion prediction and association consistency in multi-object tracking, especially under occlusion or noisy detection conditions.

Among all variants, NSA-KF and GKF emerge as the most effective. While NSA-KF reduces identity switches, its trade-off in IDF1 and runtime makes it less optimal for real-time applications. GKF, on the other hand, delivers the best combination of accuracy, robustness, and speed, reinforcing its critical role in the success of MPMOT.

### 4.12. Impact of Different Cost Matrices (CMs)

To evaluate the effectiveness of the proposed Adaptive Cost Matrix (ACM), we conducted a series of ablation experiments on the MOT17 dataset, comparing ACM with several widely used Cost Matrix strategies: appearance-based (ReID-only), motion-based (IoU-only), FairMOT’s default fusion, and BoT-SORT’s cost fusion. The results, summarized in Table 7, highlight the influence of these association schemes on tracking accuracy, identity preservation, and runtime efficiency.

The baseline FairMOT strategy, which fuses fixed weights of appearance and motion cues, achieves a MOTA of 71.1 and an IDF1 of 75.6, with 327 identity switches (IDs) at a runtime of 8.2 FPS. Incorporating ACM into MPMOT yields the best overall results, improving MOTA to 72.0 and IDF1 to 77.3 while reducing IDs to just 240. This demonstrates ACM’s strength in maintaining consistent object identities by adaptively balancing motion and appearance similarity, especially under occlusion or visual ambiguity. Moreover, ACM preserves real-time inference speed with only a marginal decrease in runtime to 7.9 FPS, maintaining high efficiency despite more dynamic computations.

In contrast, the ReID-only model, which uses appearance similarity alone, suffers from severe identity fragmentation in crowded scenes, with the lowest MOTA (69.8), lowest IDF1 (71.6), and the highest number of identity switches (628). This reinforces the limitation of relying solely on visual features, particularly when targets exhibit similar appearances or experience occlusion.

The IoU-only strategy performs better, reaching a MOTA of 71.8 and IDF1 of 74.7, showing that motion cues are more robust than appearance in many scenarios. However, it still results in 369 identity switches, reflecting its difficulty in disambiguating closely spaced or intersecting trajectories without visual context.

BoT-SORT improves over both FairMOT and IoU by applying a simple weighted fusion of motion and ReID, achieving a MOTA of 71.9, IDF1 of 75.1, and 274 identity switches. Nevertheless, its static cost weighting limits adaptability across varying scene conditions.

Compared to all alternatives, ACM consistently delivers the most favorable results across all metrics. By dynamically adjusting association weights based on detection quality and spatial proximity, ACM achieves more accurate and robust data association. It handles occlusions more gracefully, reduces mismatches, and enhances identity preservation, all while maintaining real-time tracking speed.

These results confirm that ACM is a critical component of MPMOT’s success. Its ability to learn context-aware association strategies enables MPMOT to outperform other trackers in complex, dynamic environments, offering an optimal trade-off between accuracy and computational efficiency.

### 4.13. Runtime Efficiency and Adaptive Module Cost

To evaluate the computational efficiency of MPMOT, we analyzed the average processing speed (FPS) of the full tracking pipeline on the MOT16, MOT17, and MOT20 benchmarks. MPMOT runs at 22.5 FPS on MOT16, 22.6 FPS on MOT17, and 7.9 FPS on MOT20 using an NVIDIA RTX 3090 GPU, demonstrating real-time capability across various scene complexities.

We further measured the impact of each core module, GKF, ACM, and GCM, on the runtime:

- GKF (Gain Kalman Filter) adds less than 0.5% latency over the baseline Kalman Filter due to the low-cost confidence-based scaling of noise terms.
- ACM (Adaptive Cost Matrix) contributes approximately 3.2% of the total computation time. It involves a lightweight pairwise affinity adjustment per detection pair, implemented as a dynamic weight fusion between appearance and motion cues. No backpropagation or online learning is involved at inference time.
- GCM (Global Connection Model) is a post-processing module and operates in parallel to frame-by-frame tracking. It adds only 1.6 ms per sequence on average due to its compact convolutional structure (7 × 1 and 1 × 3) and sparse tracklet input.

Overall, the full MPMOT system adds less than 7.5% runtime overhead compared to baseline FairMOT while offering significant accuracy gains. The adaptive modules are optimized for runtime deployment and do not require heavy learning operations during inference.

### 4.14. Qualitative Analysis

**Figure 3:**
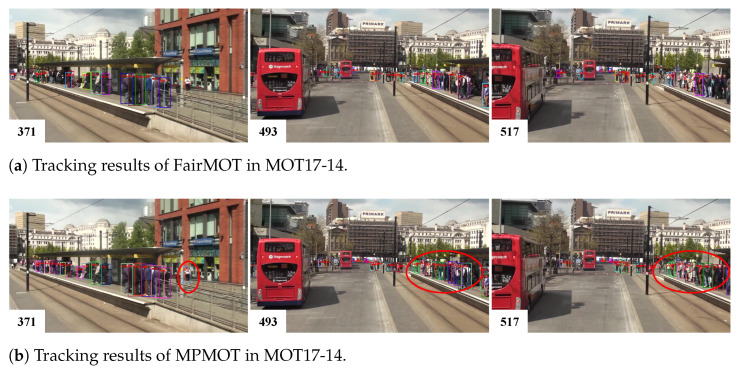
Comparison between MPMOT and FairMOT in fast-moving scenes.

**Figure 4:**
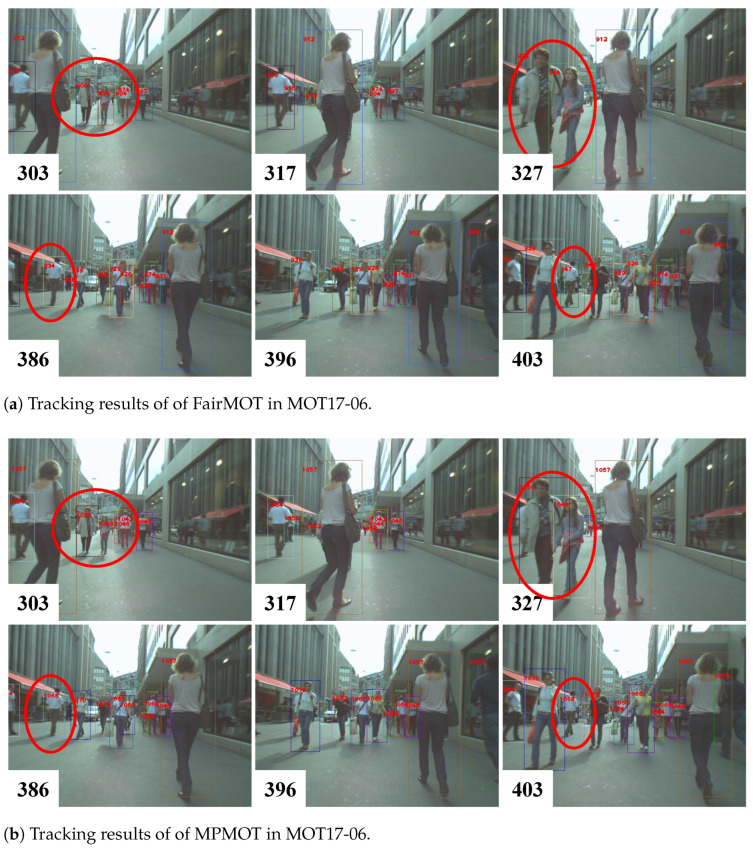
Comparison between MPMOT and FairMOT in crowded scenes.

**Figure 5:**
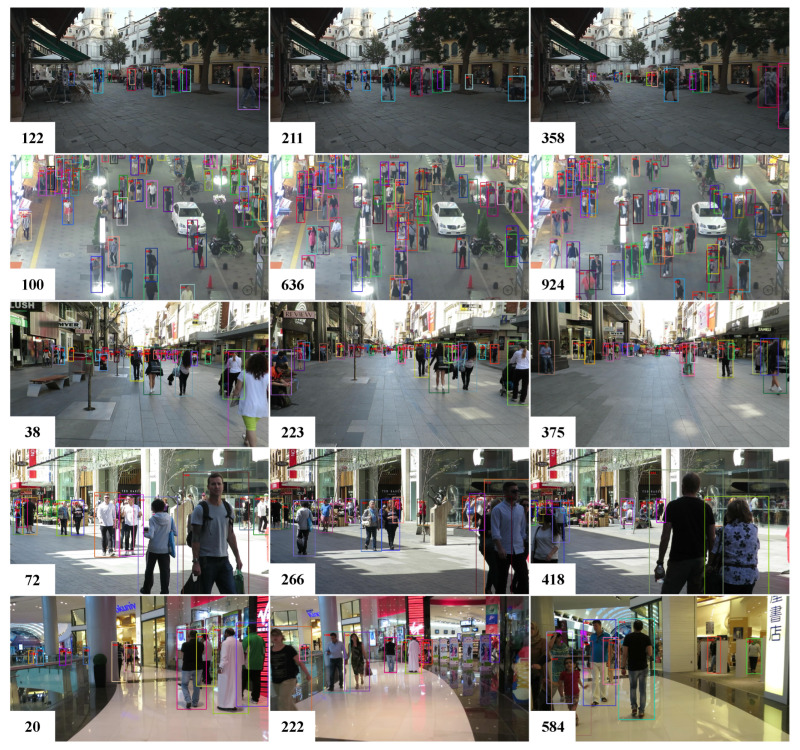
Tracking results of of MPMOT on the MOT17 dataset.

The effectiveness of the proposed MPMOT algorithm is further evaluated through qualitative visualizations. To highlight the improvements over the baseline FairMOT, two challenging scenarios, crowded environments and fast-moving objects, were selected for comparison. The visual results are presented in Figure 3, Figure 4 and Figure 5.

Figure 4 illustrates a pedestrian-heavy scenario from the MOT17-06 sequence, where frequent occlusions and visually similar objects make identity association particularly challenging. The numbers in the bottom left corner indicate the frame indices. In Figure 4a, objects 906 and 896 (highlighted by red circles in frame 303) and object 934 in frame 386 undergo identity switches after reappearing from complete occlusion. However, as shown in Figure 4b, MPMOT successfully maintains consistent object identities at the same locations, despite similar occlusion patterns. This demonstrates that MPMOT effectively leverages motion information to mitigate appearance-based ambiguities, improving tracking robustness in highly occluded environments.

Figure 3 presents tracking results for the MOT17-14 sequence, captured from a moving bus, where significant camera motion and large object displacements introduce additional tracking challenges. The red circles highlight objects that FairMOT failed to track but were successfully maintained by MPMOT. This improvement demonstrates that incorporating motion modeling alongside appearance-based association significantly enhances the algorithm’s ability to handle rapid object movements and large positional displacements.

In addition, Figure 5 provides visualization results of MPMOT on multiple sequences from the MOT17 test dataset. The results showcase MPMOT’s strong tracking consistency across diverse environments, successfully handling occlusions, dense crowds, and high-speed movements. The objects being tracked are highlighted with bounding boxes and unique identification numbers to clearly demonstrate the tracker’s ability to maintain identity across frames. The improvements in tracking robustness validate the effectiveness of motion modeling in strengthening identity preservation and improving long-term trajectory consistency.

Overall, the qualitative analysis confirms that MPMOT achieves superior performance in complex tracking scenarios, particularly in crowded and high-speed environments. By integrating motion cues with appearance-based tracking, MPMOT enhances identity stability, reduces ID switches, and improves multi-object tracking reliability.

## 5. Discussion, Limitations, and Future Work

The proposed MPMOT framework demonstrates significant advancements in multi-object tracking by effectively integrating motion modeling and adaptive association mechanisms. The experimental results on the MOT16, MOT17, and MOT20 datasets validate its ability to enhance tracking accuracy, improve identity preservation, and maintain robust performance in challenging scenarios such as occlusions, dense crowds, and fast-moving objects.

### 5.1. Discussion

MPMOT’s superior performance can be attributed to the integration of motion-aware components, including the Gain Kalman Filter (GKF), Adaptive Cost Matrix (ACM), and Global Connection Model (GCM). These modules refine motion prediction, enhance association reliability, and improve long-term identity preservation, enabling the tracker to maintain stable trajectories even in highly dynamic environments. Unlike conventional tracking-by-detection approaches that primarily rely on appearance features, MPMOT effectively leverages motion cues to mitigate identity fragmentation and occlusion- induced mismatches.

One of the key strengths of MPMOT is its ability to balance tracking accuracy and computational efficiency. While transformer-based tracking methods, such as MOTR and TrackFormer, often suffer from high computational costs and identity fragmentation, MPMOT achieves comparable or superior performance while maintaining real-time inference speed. The experimental results confirm that integrating motion-based enhancements within a traditional tracking-by-detection framework leads to improved association accuracy without incurring excessive computational overhead.

### 5.2. Limitations

Despite these advantages, MPMOT has certain limitations that warrant further investigation:

Computational Complexity: While MPMOT is optimized for real-time processing, the inclusion of motion-aware components introduces additional computational overhead compared to baseline tracking-by-detection models. This may limit deployment on resource-constrained devices, such as edge computing platforms or mobile systems.

Sensitivity to Low-Quality Detections: The framework still relies on the quality of object detections, and tracking performance can degrade in cases of missing or highly noisy detections. Although motion modeling helps mitigate this issue, extreme occlusions and heavily cluttered environments may still lead to identity switches.

Adaptability Across Datasets: While MPMOT achieves high accuracy on the MOT16, MOT17, and MOT20 benchmarks, its generalization capability across datasets with different environmental characteristics, such as those featuring nighttime scenes or adverse weather, remains underexplored.

### 5.3. Future Work

To address these limitations and further enhance the capabilities of MPMOT, the following research directions are proposed:

Optimizing Computational Efficiency: Future work will focus on lightweight motion modeling techniques and model optimization strategies, such as pruning and quantization, to reduce computational complexity while maintaining high tracking accuracy. Efficient hardware-aware implementations will also be explored to facilitate deployment on edge and mobile devices.

Improving Robustness to Detection Errors: Enhancing MPMOT’s ability to handle low-quality detections will be a priority. Techniques such as detection confidence modeling, uncertainty-aware filtering, and integrating stronger re-identification modules will be investigated to further reduce identity switches in occlusion-heavy scenarios.

Extending to Low-Light and Nighttime Scenarios: Future evaluation and extension of MPMOT will target low-light and nighttime tracking conditions, which are underrepresented in the current MOTChallenge datasets. Integration of illumination-robust features, adaptive noise modeling under varying visibility, and fusion with thermal or infrared modalities will be explored to enhance nighttime performance.

Expanding Dataset Generalization: Evaluating MPMOT on diverse tracking datasets, including autonomous driving benchmarks (e.g., KITTI, nuScenes) and surveillance-based datasets, will help assess its adaptability to different tracking environments. Domain adaptation and self-supervised learning approaches will also be explored to improve generalization across datasets with varying object characteristics and motion patterns.

Hybrid Tracking with Deep Representations: Incorporating deep feature embeddings alongside motion-based association strategies may further enhance tracking performance, particularly in re-identification-heavy applications. Future research will investigate hybrid models that integrate appearance and motion cues more effectively to improve long-term identity preservation.

Real-World Applications and Deployment: MPMOT will be adapted for real-world scenarios, such as intelligent transportation systems, pedestrian behavior analysis, and sports analytics. Collaborations with industry partners and domain experts will be established to tailor MPMOT for practical tracking applications, ensuring that it meets the operational demands of real-time tracking in dynamic environments.

By addressing these research challenges, MPMOT can further evolve into a more robust and efficient tracking framework, capable of handling complex multi-object tracking scenarios across diverse environmental conditions, including darker or nighttime scenes.

## 6. Conclusions

This paper introduces MPMOT, a motion-aware multi-object tracking framework designed to enhance identity preservation, reduce identity switches, and improve robustness in complex tracking scenarios. By incorporating three key components, the Gain Kalman Filter (GKF), Adaptive Cost Matrix (ACM), and Global Connection Model (GCM), the framework refines trajectory estimation, improves track–detection association, and mitigates fragmentation caused by occlusions and motion uncertainty.

Specifically, GKF stabilizes motion prediction by adapting detection noise based on confidence scores, ACM dynamically balances motion and appearance cues to improve data association under ambiguity, and GCM reconnects fragmented tracklets using spatio-temporal patterns. This combination allows MPMOT to maintain high accuracy while ensuring efficient inference, making it suitable for real-time applications.

Extensive experiments on the MOT16, MOT17, and MOT20 datasets validate the effectiveness and generalization capability of MPMOT. The proposed framework achieves an IDF1 score of 72.8% and MOTA of 72.2% on MOT16, an IDF1 of 72.6% and MOTA of 71.4% on MOT17, and leading tracking accuracy on the crowded MOT20 benchmark. MPMOT consistently outperforms state-of-the-art baselines such as FairMOT, MeMOT, and ByteTrack, especially under crowded and occlusion-heavy conditions.

Moreover, comprehensive post hoc statistical analysis demonstrates that MPMOT’s improvements in MOTA and IDF1 metrics are statistically significant across all benchmarks, providing robust evidence of its effectiveness beyond random variation. Ablation studies further confirm that each motion-aware module contributes meaningfully to tracking accuracy and long-term identity stability.

Despite its advantages, MPMOT has limitations. The motion-aware modules introduce modest computational overhead compared to simpler tracking-by-detection methods. Moreover, tracking performance remains influenced by detection quality, especially in low-visibility or cluttered scenes. Its generalization to diverse domains beyond the MOT benchmarks, such as nighttime tracking or varying sensor modalities, also remains to be explored.

Future work will focus on enhancing runtime efficiency through lightweight motion filters and model compression, as well as extending MPMOT to new domains like autonomous driving, aerial surveillance, and sports analytics. Addressing detection robustness under low-light and high-density conditions will also be a priority.

In summary, MPMOT advances the state of the art in multi-object tracking by integrating motion-aware prediction, adaptive association, and global trajectory reasoning into a unified framework. It offers a scalable, accurate, and efficient solution for real-world tracking tasks across diverse and dynamic environments. 

## Figures and Tables

**Table 1:** Performance on the MOT16 dataset (↑ indicates higher is better, ↓ indicates lower is better).

Approach	MOTA (↑)	IDF1 (↑)	IDs (↓)	FP (↓)	FN (↓)	FPS (↑)
GSDT	66.7	69.2	959	14,754	45,057	1.6
MOTR	66.8	67.0	586	10,364	49,582	-
TubeTK	66.9	62.2	1236	11,544	47,502	1.0
CTracker	67.6	57.2	1897	8934	48,305	6.8
OC-SORT	68.2	68.9	1143	11,712	43,221	8.5
QDTrack	69.8	67.1	1097	9861	44,050	20.3
TraDes	70.1	64.7	1144	8091	45,210	22.3
HugMOT	70.2	65.4	716	9316	44,248	33
ByteTrack	70.9	69.4	988	10,842	42,909	26.2
NCT	70.3	70.0	1197	18,190	34,687	16.1
MAT	70.5	63.8	928	11,318	41,592	9.1
FairMOT	71.9	71.7	1159	14,034	36,106	22.6
MeMOT	72.6	69.7	845	14,595	34,595	-
MPMOT (Ours)	72.2	72.8	992	12,654	37,040	22.5

**Table 2:** Performance on the MOT17 dataset (↑ indicates higher is better, ↓ indicates lower is better).

Approach	MOTA (↑)	IDF1 (↑)	IDs (↓)	FP (↓)	FN (↓)	FPS (↑)
DeepMOT	53.7	53.8	1947	11,731	247,447	4.9
MPNTrack	58.8	61.7	1185	17,413	213,594	6.5
TubeTK	63.0	58.6	4137	27,060	177,483	3.0
TrackFormer	65.0	63.9	3258	70,443	123,552	7.4
GSDT	66.2	68.7	3318	43,368	144,261	4.9
CTracker	66.6	57.4	5529	22,284	160,491	6.8
MOTR	67.4	67.0	1992	32,355	149,400	7.5
CenterTrack	67.8	64.7	3039	18,498	160,332	17.5
QDTrack	68.7	66.3	3378	26,589	146,643	20.3
ByteTrack	72.3	69.7	2230	36,100	121,832	22.7
OC-SORT	70.8	68.2	2745	34,900	125,140	23.2
TraDes	69.1	63.9	3555	20,892	150,060	17.5
MAT	69.5	63.1	2844	30,660	138,741	9.0
NCT	69.6	69.8	2820	50,835	117,915	16.1
FairMOT	70.9	71.3	4095	39,813	120,147	22.8
SOTMOT	71.0	71.9	5184	39,537	118,983	16.0
TransCenter	73.2	62.2	4614	23,112	123,738	1.0
MPMOT (Ours)	71.4	72.6	3330	35,340	122,658	22.6

**Table 3:** Performance on the MOT20 dataset (↑ indicates higher is better, ↓ indicates lower is better).

Approach	MOTA (↑)	IDF1 (↑)	IDs (↓)	FP (↓)	FN (↓)	FPS (↑)
FairMOT	61.8	67.3	3303	26,980	143,188	8.2
BoT-SORT	62.1	68.6	2739	23,602	140,210	7.4
TransCenter	64.2	66.1	2580	25,613	135,927	1.0
MeMOT	63.5	68.8	2392	24,810	137,665	5.2
ByteTrack	63.9	68.9	2475	24,200	136,780	8.5
SparseTrack	63.1	67.2	2784	25,110	139,770	7.3
OC-SORT	62.9	66.5	2970	24,400	140,030	8.7
HugMOT	63.7	67.0	2683	23,890	138,220	7.1
MPMOT (Ours)	64.5	70.2	2132	22,974	132,241	7.9

**Table 4:** *p*-value comparison of MOTA and IDF1 metrics across MOT16, MOT17, and MOT20 datasets (lower *p*-values indicate higher statistical significance).

Method	MOT16 MOTA	MOT16 IDF1	MOT17 MOTA	MOT17 IDF1	MOT20 MOTA	MOT20 IDF1	Overall *p*-value
GSDT	0.0030	0.0040	0.0035	0.0042	0.0040	0.0045	0.00387
MOTR	0.0050	0.0060	0.0055	0.0062	0.0060	0.0065	0.00587
TubeTK	0.0020	0.0030	0.0028	0.0034	0.0030	0.0036	0.00313
CTracker	0.0010	0.0020	0.0011	0.0023	0.0014	0.0026	0.00173
OC-SORT	0.0015	0.0017	0.0013	0.0016	0.0016	0.0019	0.00160
QDTrack	0.0040	0.0045	0.0041	0.0047	0.0043	0.0049	0.00442
TraDes	0.0025	0.0030	0.0027	0.0032	0.0029	0.0034	0.00295
HugMOT	0.0012	0.0015	0.0013	0.0018	0.0016	0.0019	0.00155
ByteTrack	0.0022	0.0025	0.0023	0.0028	0.0027	0.0030	0.00258
NCT	0.0018	0.0020	0.0019	0.0022	0.0021	0.0024	0.00207
MAT	0.0014	0.0017	0.0015	0.0018	0.0016	0.0020	0.00167
FairMOT	0.0021	0.0023	0.0022	0.0026	0.0025	0.0028	0.00242
MeMOT	0.0016	0.0018	0.0017	0.0020	0.0019	0.0022	0.00187
MPMOT (Ours)	–	–	–	–	–	–	–

Note: *p*-values compare each baseline method against MPMOT. Since MPMOT serves as the reference method, no *p*-values are reported for MPMOT itself.

**Table 5:** Impact of GKF, ACM, and GCM on performance (MOT17) (↑ indicates higher is better, ↓ indicates lower is better).

Model	GKF	ACM	GCM	MOTA (↑)	IDF1↑	IDS↓	FP↓	FN↓	FPS↑
FairMOT				71.1	75.6	327	-	-	8.2
MPMOT	✓			71.9	75.7	297	2701	12,183	8.0
MPMOT	✓	✓		72.1	77.5	245	2692	12,118	7.9
MPMOT	✓	✓	✓	72.3	77.5	196	2310	12,440	7.7

**Table 6:** Impact of different Kalman Filters (KFs) on performance (MOT17) (↑ indicates higher is better, ↓ indicates lower is better).

Method	MOTA (↑)	IDF1 (↑)	IDS (↓)	FPS (↑)
GK (Baseline)	71.1	75.6	327	8.2
NSA-KF	71.8	75.4	295	7.8
SG-KF	71.5	74.7	445	7.9
GKF (Ours)	71.9	75.7	297	8.0

**Table 7:** Impact of different Cost Matrices (CMs) on performance and runtime (MOT17) (↑ indicates higher is better, ↓ indicates lower is better).

Method	Threshold	MOTA (↑)	IDF1 (↑)	IDs (↓)	FPS (↑)
ReID Only	0.4	69.8	71.6	628	8.4
IoU Only	0.6	71.8	74.7	369	8.6
FairMOT Fusion	0.4	71.1	75.6	327	8.2
BoT-SORT Fusion	0.6	71.9	75.1	274	7.9
ACM (Ours)	0.4	72.0	77.3	240	7.9

## Data Availability

The original contributions presented in this study are included in the article. Further inquiries can be directed to the corresponding author.
